# Epidemiology of Developmental Dysplasia of Hip in Pakistan: Insights from the Paediatric Orthopaedic Registry Pakistan (PORP)

**DOI:** 10.12669/pjms.41.3.10922

**Published:** 2025-03

**Authors:** Anisuddin Bhatti, Marium Habib Soomro, Muhammad Amin Chinoy, Atiq Uz Zaman, Muhammad Aslam Baloch, Pervez Ali, Mansoor Ali khan, Umair Nadeem, Muhammad Badaruddin Zafir, Muhammad Jamil, Asif Peracha, Mehtab Ahmed Pirwani, Zakiuddin Ahmed

**Affiliations:** 1 Anisuddin Bhatti, FCPS Consultant Orthopaedic, Trauma & Paediatric Orthopaedic Surgery, Neurospinal Cancer Care Institute, Karachi, Pakistan. Professor, Dr. Ziauddin University Hospital, Clifton Karachi, Pakistan; 2 Marium Habib Soomro, MSPH General Manager, Health Research, Advisory Board (Health RAB) Karachi, Pakistan; 3 Muhammad Amin Chinoy, FRCS Professor, Indus Hospital Karachi, Pakistan; 4 Atiq Uz Zaman, FCPS Professor, Gurki Trust Teaching Hospital, Lahore, Pakistan; 5 Muhammad Aslam Baloch, FCPS Associate Professor, Sheikh Khalifa Bin Zayyad Hospital Quetta, Pakistan; 6 Pervez Ali, FCPS Associate Professor, Jinnah Postgraduate Medical Centre, Karachi, Pakistan; 7 Mansoor Ali khan, FCPS Professor, Agha Khan University Hospital, Karachi, Pakistan; 8 Umair Nadeem, FCPS Assistant Professor, Gurki Trust Teaching Hospital Lahore, Pakistan; 9 Muhammad Badaruddin Zafir, FCPS Assistant Professor, Nishtar Medical University Hospital Multan, Pakistan; 10 Muhammad Jamil, FCPS Assistant Professor, DIMC Ojha Campus DUHS Karachi, Pakistan; 11 Asif Peracha, FCPS Assistant Professor, Liaquat National Hospital Karachi, Pakistan; 12 Mehtab Ahmed Pirwani, FCPS Consultant, Fatimiyah Hospital Karachi, Pakistan; 13 Zakiuddin Ahmed, MBBS, EMBA General Secretary, Health Research Advisory Board (Health RAB)

**Keywords:** Developmental Dysplasia Hips, DDH, Demographics, Risk factors, Complications, Topographic Distribution, DDH Outcomes

## Abstract

**Objective:**

This study aims to determine prevalence, risk factors and geographic distribution of Developmental Dysplasia of the Hip in Pakistan and to assess the cumulative outcomes of various treatments used and propose recommendations to establish preventive strategies and best treatment practices in Pakistan.

**Methods:**

This multicentric retrospective study was conducted with analysis of data from DDH section of PORP registry of Pakistan. The data was uploaded by 1,3-11 authors, which were treated during last three decades. The evaluation parameters included 25 variables of basic demographics of patients, geographic prevalence, characteristics of DDH and related risk factors. The data was also analyzed to know methods of treatment used and cumulative outcomes in various age groups and severity of dysplasia.

**Results:**

The study included 755 patients with 1,107 affected hips, aged one day to over eight years. Of these, 86.25% were diagnosed after 18 months, 11.7% were over eight years. Among 104 neonates, 23% had neonatal screening. The female-to-male ratio was 3:1. 21% had history of DDH in family, and 24% were from remote rural areas. 46.6% had bilateral DDH. 48.43% patients had significantly obtuse acetabular index >45°. The acetabular index (AI) found highly associated with age bilaterally (p=0.001). 88% of normal unilateral hips had AI <30°, and 11% have moderate dysplasia of 30°-45°. 129 patients were treated non-operatively with 83.72% success rate at minimum three years follow-up. The failure rate of Pavlik harness was 25%. Six hundred twenty six (626) patients underwent open reduction with 70.42% success rate at minimum one year follow-up. Most failures in conservative and operative treatment were on one side of bilateral cases. Late complications over 10 years follow-up was short femoral neck offset, coxa magna and residual acetabular dysplasia.

**Conclusion:**

This study leverages PORP registry data to identify DDH demographics, risk factors, and treatment outcomes. It highlights the need for establishment of MSK screening protocols, to diagnose DDH at earliest, to prevent development of disability of late treatment and enhance best practices in DDH management.

## INTRODUCTION

Developmental Dysplasia of the Hip (DDH) is a common developmental musculoskeletal deformity that goes undetected without early musculoskeletal (MSK) screening. In countries with established neonatal screening programs, late-presenting & missed dislocations are rare, affecting less than 1.4% to 34% of the study population.^1^,^2^ However, in low- and middle-income countries late presentations are more frequent due to less practiced MSK screenings and often necessitating more invasive treatments.^3^,^4^ The success rates for DDH treatment significantly decline with age, from 95% before age two to as low as 42% after age eight years.^3^ These findings underscore the importance of early referrals, diagnosis, and treatment.

The Pakistan Bureau of Statistics (PBS) census 2017 report identify 913,667 disabled individuals, 40% of whom are children, with 69.89% living in rural areas.^5^ However, classified Paediatric MSK disabilities has never been identified. To address this issue, the Paediatric Orthopaedic Society of Pakistan (POSP) launched the Paediatric Orthopaedic Registry of Pakistan (PORP) on September 30, 2021, with a milestone slogan of “Born with deformity! Why to live with disability.” The registry tracks various Paediatric MSK issues, including DDH and Clubfoot.

The study aimed to examine the prevalence, demographics, risk factors, and general characteristics of DDH. Also to reveals how socio-economic factors and healthcare access contribute to delayed presentations and affecting treatment outcomes. This study shall help in establishment of effective MSK screening protocols, preventing disabilities with early referrals to enhance best practices in DDH management.

## METHODS

This retrospective multicentric study analyzed data from the DDH section of the PORP registry, covering 755 patients with 1107 affected hips out of 806 cases uploaded in PORP till 30^th^ September 2023. These 755 patients were managed by 10 contributors (authors 1, 3-11) between 1^st^ January, 1993 to 30^th^ September 2023, inclusive retrospective entries of patients managed by contributors (ANB, MAC, MAK) from 1^st^ January, 1993 till 1^st^ October 2021.

### Ethical Approval:

The study was approved by the Institutional Research Review Committee of the Neuro-Spine Cancer Care Institute Karachi (No. F.NCCI/ICR 2023/17102023, Date: November 30, 2023), the signatory of the MOU with the PORP steering committee.

Data was entered by contributors using a web-based, responsive software platform (https://www.porp-registry.pk/) hosted on a cloud server financed by PharmEvo Pakistan & technically supported by Health Research Advisory Board (HealthRAB). Each contributor is provided a password-protected individual login. Participants restricted to viewing only their own data. However, the Director and Coordinator of PORP have access to the entire dataset, to download and use for publication of annual report, that as allowed by MOU by contributors with the PORP steering committee. The software includes a data export feature, enabling easy downloading of data in Excel format. This study used only 25 variables related to patients demographics, geographic distribution, DDH characteristics of laterality, severity of dislocation and acetabular index. DDH related risk factors of Family history, associated deformities, screening protocols received, position at birth were also included. The treatment modalities used and cumulative outcome were included as well. The study excluded patients uploaded in PORP by two out of 24 contributors who entered only two cases that too as test entries, with deficient data. The personal password-protected data of individual authors’ for outcomes were excluded. Based on severity of dysplasia, the patients were categorized into three groups of the acetabular index (AI), four groups of Tonnis height of dislocation and five age groups to evaluate treatment outcomes. The outcomes were assessed using Bhatti’s Functional Hip Score,^6^ Mackay’s clinical criteria, and Severin’s radiologic grading.^3^,^4^,^6^ The minimum follow-up for evaluation was set at one year, with successful outcomes defined as excellent to good, and unsuccessful outcomes as fair to poor.

### Data Analysis:

The database was developed and analyzed by SPSS version 23. Age of patients were converted into five groups and presented by frequencies. Gender classified into male and female, laterality presented as unilateral and bilateral and presented by their frequencies. To assess the association of age with gender and laterality, Chi-square test of independence was employed. Association of Acetabular Inclination of affected hips with DDH & normal hips were also determined by Chi-square test of independence. The results were considered significant at p<0.05.

## RESULTS

Patient ages ranged from 1 day to over 8 years, with an average age of four years (±3.8 years). The majority (74.43%), were diagnosed during or after the walking stage, while 11.7% were diagnosed after age eight, and 13.7% were neonates (Table-I). Of these newborns, 23% were detected via neonatal screening. Girls were in majority (72.6%), with a female-to-male ratio of 3:1, particularly prominent in those over three years old. Unilateral DDH affected 53.4% patients, while bilateral dislocations were more common among late presenters (Table-I). Gender showed statistically significant distribution among different age groups (p=0.01) and Laterality did not depend upon age groups (P=0.69).

**Table-I:** Age, Gender and Laterality Correlation (N-755).

Gender	Age Groups						Percent %
	0-11 Months	12-18 Months	19-35 Months	3-8 Years		9 Years & Above	
Female	68	72	87	254		67	548 (72.6%)
Male	36	43	31	75		22	207 (27.4%)
*Statistically significant distribution among Gender vs different age group found (p=0.01)*							
*Laterality*							
Bilateral	48	59	50	156		39	352 (46.6%)
Unilateral:	56	56	68	173		50	403 (53.4%)
*Right-240 And Left-163. Laterality revealed statistically no dependence upon age groups (P=0.69).*						
Total Patients	104(13.7%)	115 (15.2%)	118 (15.6%)	329 (43.5%)		89 (11.7%)	755 (100%)

**Fig.1:**
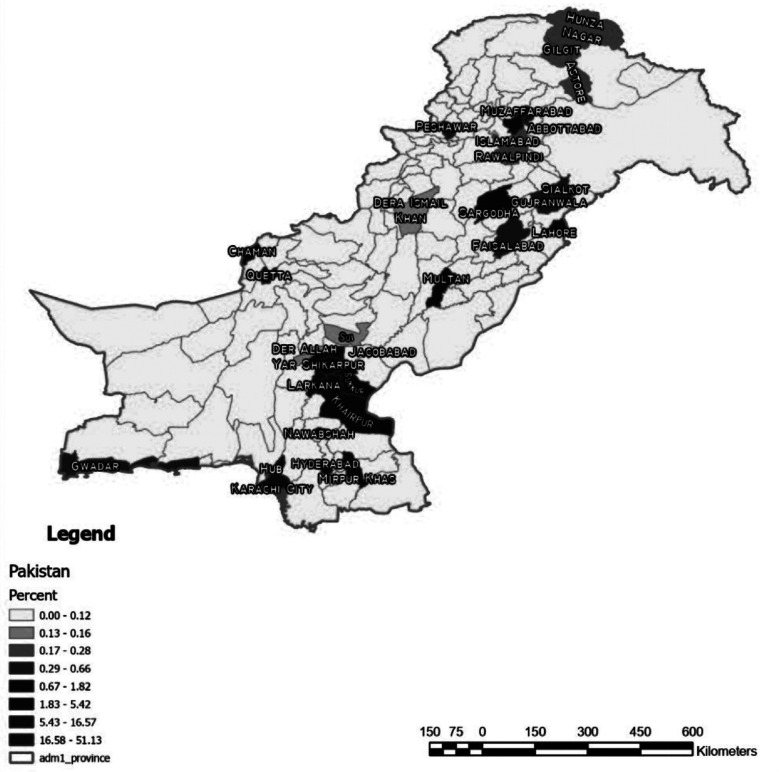
Geographic Mapping of the patient’s residence (N-511).

Out of declared 683/755, the most patients (71%) were from Sindh province, 17% from Baluchistan, 9% from Punjab, 0.5% from Khyber Pakhtunkhwa, and 2.3% from Gilgit-Baltistan and Azad Jammu & Kashmir. A small number (0.52%) were from Afghanistan. The high percentage from Sindh corresponds with the fact that 71% of data entries originated from this province, that also include permanent residents of KPK, Baluchistan and Northern areas. Geographic mapping (Fig.1) revealed, 24% of patients from remote rural areas, where most births occurred at home, 57%% were from cities and nearby villages with limited healthcare access, and only 23% born in teaching hospitals that provided MSK screening.

Family history of DDH was reported by 32% of 243 declared entries. Fetal position at birth was declared in 521 cases with Breech in 30.13% and vertex in 69.86%. Additionally, 18.80% (142 out of 755) had associated risk factors, including clubfoot deformity (4.6%), torticollis (4.3%), pes plano valgus (1.6%), radial clubhand (1.2%), and congenital pseudoarthrosis of the tibia (0.7%). Furthermore, 6.75% of DDH cases were linked to syndromes such as AGMC (5.3%), cerebral palsy (0.3%), hyperlaxity syndrome (0.92%), and spina bifida (0.3%). Hyperlaxity was typically diagnosed late, often following relapse or re-dislocation.

Among 1,107 hips affected by DDH, Tonnis classification was declared in 975 hips. Of these, majority 59% had Tonnis IV and 30% had Tonnis III dislocations. The acetabular index (AI) was recorded for 927 hips (83%), with 48.43% having significantly vertical AI (>45°). The acetabular index found highly associated with age bilaterally (p=0.001), while side of hips vs AI angles was not significant (p=0.43). (Table-II). Of 403 normal hips in unilateral DDH, AI was declared in 60%, with majority (88%) having nearly normal AI <30°, and 11% have moderate dysplasia of 30°-45°.

**Table-II:** Acetabular Inclination in DDH affected hips (N-927).

AI Declared (Right)	Age Groups vs Right side					Total
	0 -11 Months	12 -18 Months	19 -35 Months	3-8 Years	9 years & above	(%)
AI<30°	26	21	34	55	9	145
AI=30° - 45^o^	19	20	11	35	9	94
AI>45^o^	8	42	47	111	28	236
Total	53	83	92	201	46	475
Chi-square=36.57, p=0.001 significant.						
*AI Declared (Left)*	*Age Groups vs left side*					*Total %*
	*0 -11 Months*	*12 -18 Months*	*19 -35 Months*	*3-8 Years*	*9 years & above*	
AI<30^o^	16	24	24	49	10	123
AI=30^o^ - 45^o^	22	21	16	45	12	116
AI>45^o^	7	31	53	98	24	213
Total	45	76	93	192	46	452

Chi-square=27.96 , p=0.001 significant

Among 60 patients under seven months age were treated with Hip Abduction Braces (HAB), 11.66% of Pavlik Harnesses (PH) needed conversion to fixed abduction braces due to instability. The bracing failure rate was 25% (15/60), mainly involving unilateral failure in bilateral DDH. All these failed containment patients were initially managed with closed reduction but eventually required open reduction. About 3.33% of these were operated through medial approach and others 5% through the Smith-Petersen approach. Whereas, 8.69% patients of 7-18 months age, initially treated with Closed Reduction & Spica cast (CR-SC) also needed open reduction. Overall satisfactory outcomes among patient with conservative treatment was 83.72% at a 3-year follow-up. Two cases of this group had complication of short neck in one and another had coxa magna at over 10 years follow-up.

Open reduction and capsulorraphy (ORC) were performed in 83.57% of patients including five patients of age group 10-11 months. (Table-III). Eleven patients of this group initially received PH (5) & CR-SC (6) treatment elsewhere. The pelvic osteotomies (PO) were performed in 406 patients. Of these, Salter’s osteotomies comprised 63.79%, peri-acetabular-Pemberton-Dega (32%), Chiari’s (2.21%), and Triple Pelvic Osteotomies (TPO) in 1.97%. The most (80.29%) of the Salter’s and modified Salter’s osteotomies were performed on those over three years of age, while 19.70% were under three years. Chiari’s osteotomy and TPO were mostly done in patients over eight years of age (Table-III).

**Table-III:** Open reduction of DDH in various age groups facilitated with Femoral and Pelvic Osteotomies (N-631).

Procedures in patients	Age groups					Total N-631
	0-11 Months (N-104)	12-18 Months (N-115)	19-35 Months (N-118)	3-8 Years (N-329)	9 Years & Above (N-89)
*Single incision approach, age group <3 years (N=176)*						
OR Medial Ap	2	x	x	x	x	2
ORC	3	22	8	46	9	88***
ORC + PO	0	55	10	0	0	65
ORC + DFDRO	0	6	0	0	0	6
ORC + DFDR+PO	0	7	8	0	0	15 (176)
*Double incision approach, age group >3 years (N=455)*						
ORC + PFSDRO	0	0	60	52	17	129
ORC+ PFSDRO+PO	0	0	32	231	63	326 (455)
Total ORC	5	90	118	329	89	631
*** *Osteotomies either not done or not declared.*						

Ap= Approach., ORC= Open Reduction and Capsulorraphy., PO=Pelvic osteotomy., DFDRO= Distal Femoral Derotation Osteotomy., PFSDRO= Proximal Femoral Shortening and Derotation Osteotomy.

The satisfactory outcomes were achieved in 70.42% of patients at minimum follow-ups of one year and more. Follow-up durations varied, with 61.85% lasting 1-3 years and 11.73% exceeding 16 years. Complications occurred in 7.67% of cases, including re-subluxations or re-dislocations in 4%, deep infections in 1.38%, avascular necrosis (AVN) in 1.12%, and fibrous ankylosis in 1.12%. Long-term issues (after age 10 years) included uncovered femoral head in 2.9%, short femoral neck in 1.35%, deformed femoral head in 2.7%, and limb length discrepancy (LLD) in 1.12% and two of them had limb lengthening with Illizarov technique.

## DISCUSSION

Developmental Dysplasia of the Hip (DDH) represents the most common developmental disorder of MSK system of the infant and that remain obscure unless detected early with clinical and ultrasonographic evaluation.^2^,^7^-^9^ The incidence reported in various countries around the world range widely between 0.4% and 35% in live births.^2^,^7^ The recent (2017-2024) systematic reviews and meta-analysis from developed world and our neighborhood of the China,^7^ India,^9^ Turkey^10^ and Iran^8^,^11^ reports widely ranged incidence from 25.8% to 47.99% among infants with risk factors compared to 3.21% to 2.8% in those without risk factors.^2^,^7^-^13^ Whereas, incidence of missed and late diagnosed DDH significantly decreased from 34% to 0.9%^2^,^13^ with better child rearing practice and preventive strategies of universal clinical examination and selective (risk based) ultrasound screening, and genetic counseling.^1^,^2^,^7^-^13^ Hattori et al.^2^ from Japan report 15% late diagnosed DDH at >1 year of ge, while 18% of these diagnosed very late at >3 years and majority of these were “missed dislocation”. Mulpuri et al.^1^ and Hattori et al.^2^ defined “missed dislocations” as those discovered months after a normal examination by an expert clinician,^1^ late diagnosed as after one year age and very late after three years age.^2^ However, the DDH prevalence has never been reported so far from our country, as MSK screening is not being practiced at large. Almost all our native studies report DDH in walking-age children, mostly seeking treatment after three years age.^4^,^14^-^16^ Nevertheless, PORP data, however revealed, 27% of children received neonatal screenings that too were reported from tertiary care or university hospitals. Hence, the ratio of late and too late presentations in PORP (Table-I) was significantly high (86.13%) compared to our neighbors India, Iran and Turkey.^7^-^9^,^11^ The reasons cited for high rates of late, too late and neglected DDH in our native literature includes affordability, misconceptions about surgical complications, limited treatment resources, and swaddling.^4^,^5^,^17^

Chen X et al.^7^ identified gender as the most significant risk factor for DDH with prevalence 6.97 times higher in women than men. PORP revealed a female-to-male ratio of 3:1 (p=0.01), which is also comparative with global reports of female preponderance of 2.5:1.5 to 3.5:1.^1^,^2^,^4^,^8^-^10^,^12^,^18^ Interestingly, PORP data revealed male predominance at the extremes of age, while females were more prevalent among age 18 months to seven years, similar findings were also reported by Hattori et al.^2^

The PORP revealed 32% of cases having family history of DDH, that is consistent with a range of 27% to 34.6% as reported by Japan,^2^ Turkey^10^ and China.^7^ They identified substantial difference in incidence of DDH between first degree and second degree relatives. Moreover, a single report from Khyber Pakhtoon Khuwa^19^ reports family history in 10.43%. Henceforth we can consider this range of 10.43% to 32% identified by KPK and PORP as the representing figure from Pakistan.

The risk of DDH in infants with MSK anomalies reported by a Chinese study^7^ revealed approximately double than in normal babies, whereas an Iranian study^11^ reports highest rate of DDH among overall prevalence of 27.5% MSK anomalies in infants. The PORP however revealed 18.8% patients had combined MSK anomalies, foot anomalies in 6.2% and 4.3% had associated muscular torticollis. PORP findings are consistent with 7.56% reported by Perry et al.^20^ and higher than KPK^21^ and Haberg et al.^22^ reports of 4.16% & 4.3% respectively. The MSK combined foot anomalies and torticollis has been reported in literature as possible risk factors, and strongly advocate for a selective ultrasound screening for newborns with foot abnormalities, they view congenital foot malformations as a significant factor for DDH.^8^,^10^,^18^-^20^,^22^

The vertex presentation at birth in PORP was identified in 69.1% patients and breech in 30%, that was significantly higher than KPK province reports of 20.9% breech.^21^ Whereas, studies from Japanese,^2^ Turkish^10^ and Greece^12^ identified breech positions at birth in 15% to 23%. Mulpuri K et al.^1^ reports “cephalic birth presentation and swaddling having a significant correlation with early and late presentations of DDH.

Among laterality, the PORP revealed unilateral (53.4%) dislocations, 7% more than bilateral (46.6%) (Table-I), that were consistent with native and global reports.^2^,^14^-^16^ Moreover, Right sided dislocation were more than left in PORP and Ali AM et al.^15^ report of (59% & 54%). However, PORP revealed no significant correlation of age with laterality (P=0.69). Whereas, Hattori et al,^2^ Zimri,^14^ Kotlarsky^18^ and Seringe,^23^ reported higher prevalence of left-sided (60%) DDH. The Seringe^23^ attribute left-sided predominance to the left occiput anterior position of non-breech newborns, where the hip is adducted against the mother’s spine, limiting abduction.

The PORP revealed 24% patients belongs to most remote rural areas (Fig.1), which is comparable to Iranian and Indian reports.^8^,^9^,^11^ We feel PORP percentage (24%), little ambiguous due to frequent re-settlement at Karachi from other provinces. Similarly, late and neglected presentations of DDH despite diagnosed earlier, were found higher from remote areas. The major factor attributed to this late presentation being the scarcity and insufficient health care facilities available to rural population which constitutes 63.56% of total populace, mostly observing cultural practices of consanguineous marriages and swaddling.^4^,^5^ Similar to Geographic variation in prevalence of DDH across the provinces in Pakistan, the world literature cite difference in the prevalence rate across different part of countries due to differences in genetics, racial, cultural practices of swaddling, consanguineous marriages, socioeconomic factors and methods of MSK evaluation.^2^,^4^,^7^-^9^,^11^,^17^,^18^,^23^ Most of these recommend necessity of genetic counseling especially in consanguineous marriages, and changes in wrapping technique (safe sleepwear swaddling) instead traditional swaddling, to decrease the incidence of DDH.^7^,^9^-^11^

Tonnis height of dislocations, obtusity of acetabulum (AI) and deformation of caput femoris has been seen in a direct relationship with progressively increasing age and weight bearing (Table-II). That, necessities open reduction with femoral shortening and acetabular reconstruction for children over 30 months to prevent further deformation, Avascular Necrosis and re-dislocations.^3^,^4^,^18^ The PORP revealed sever acetabular dysplasia (AI >45°) in 47.78%, while 22.65% had moderate (AI 30°-45°) acetabular dysplasia (Table-II). Among normal side of unilateral, PORP also revealed some variation in in AI, that 89.21% had an AI <30°, while 9.95% had AI >30° (Table-II). This aligns with a Turkish study by Akel & Yilmaz,^24^ who observed mean AI values of 20° -14.1° in boys and 20°-15.7° in girls in the normal hips and a decreasing trends in age groups six months to six years. PORP findings however, categorized AI into three broader groups (Table-II) that may not truly reflect precise AI. We suggest a dedicated study for the purpose to explore more precise AI in healthy children.

The treatment outcome of DDH significantly depend on age at commencement of treatment, severity of dysplasia, and patient compliance. Zadeh et al^3^ reports, age as a significant prognostic factor and the outcomes progressively decreases with increasing age, as the capacity for hip remodeling diminishes after age four and ossification of triradiate cartilage after eight years”. Hence the Pelvic osteotomies after age of 3-4 years become mandatory to avoid uncovered head at adolescent age.^3^,^4^,^16^ This was reflected in PORP data, where in open reduction with Pemberton & Salter’s PO were done in 70.99% and more aggressive Triple Pelvic Rotational Osteotomies in 11.7% patients in age over 8 years. (Table-II).

The cumulative clinical and radiologic outcomes in PORP at a three-year follow-up with Hip Abduction Brace (HAB) revealed as 75% satisfactory in <7 months age and 83.72% success rate with closed reduction casting (CR-SC) >7 months age. The overall failure rate with HAB and CR-SC in PORP was significantly higher than 10%-14% failures reported by Kotlarsky^18^ and Walton MJ.^25^ Most of these failures in PORP and Kotlarsky review^18^ were on one side of bilateral DDH.

In open reductions, the cumulative overall clinico-radiological outcome in PORP was 77% satisfactory. While age-specific analysis revealed a notable trend of increasing age at open reduction correlates with decreased satisfaction (Table-III), that reiterates the findings of other studies as well.^3^,^4^,^14^,^15^,^18^,^19^ Kotlarsky^18^ however quotes further that “treatment after eight years often leads to poorer outcomes compared to untreated cases”. The PORP findings also re-endorse the observation of Kotlarsky^18^ “that bilateral DDH treated surgically has poor outcomes, often due to initial poor reduction and re-dislocations”.

The PORP revealed a higher incidence of re-subluxation-dislocations (11.8%) compared to global literature percentage of 1.4%-8.33%.^3^,^4^,^14^,^15^,^18^ Whereas AVN (0.6%) and deep seated infection (0.3%) at lower percentage than above reports with 1.4%-8.33% and 3.3% respectively. On long-term follow-ups (over 10 years) in PORP revealed complications in 19% cases, like short neck and coxa magna in some cases, potentially due to late-onset AVN, that has also been revealed by Zadeh.^3^

## CONCLUSION

Over the past three decades, the PORP data revealed a significant paradigm shift in the age distribution of DDH cases. That, in the 1991-2010s, 71% of cases were diagnosed in children over three years age. However, from 2011 to 2020, there has been a noticeable increase in number of children under three years. This trend reflects increased interest of native orthopedic surgeons in subspecialty. Despite this progress, referrals for late presentations (age 6-7 years) have remained consistent, indicating persistent issues with early detection and treatment. Most of these late referrals were from lower & middle socio-economic backgrounds, remote areas and neglected patients influenced by misconceptions about the efficacy of DDH. The PORP findings highlighted the importance of neonatal clinical MSK evaluation and selective ultrasonographic screening in high risk patients for DDH. It will significantly decrease the incidences of late presentations and prevent development of significant problems of hip deformation despite the best treatment provided by then. The early diagnosis, timely referral and intervention significantly reduce financial burden on the country and psycho-social-economic burden on the affected families.

### Recommendations:

We strongly advocate for establishment of a neonatal MSK screening protocols and early referral system at all primary, secondary and tertiary health centers. This can be accomplished by providing teaching-training workshops for healthcare professionals including birth attending doctors, nurses and neonatologists. By providing teaching and training ultrasonographic techniques to the residents of radiology, Ob-Gynae, Orthopaedic and Paediatric surgery and to develop Guidelines for DDH screening protocols with reference to available guidelines in literature.^12^ The program guidelines can be developed by taking help from one of existing sustainable model of clubfoot treatment in Pakistan which was published by Ponseti International in 2015. The “Clubfoot disability: Model for Sustainable Health System programs in three countries; Peru, Pakistan and Nigeria”,^26^ that signifies remarkable achievement including establishment of 148 Clubfoot Clinics across Pakistan, also referring to its impact on getting earliest referrals soon after births.

### Limitations:

It includes deficient data entries on patient ethnicity, original residence, neonatal screening, risk factors, and associated disorders. Inadequate documentation of procedural details, such as previous surgeries and type of osteotomies, and incomplete outcome data further compromised the study’s comprehensiveness. Moreover, PORP figure may not be an accurate reflection of DDH epidemiology in Pakistan, as this number is a reflection of participating surgeons instead a national survey.

### Strengths:

Despite these limitations, the PORP study shall be a valuable resource for native and international literature. The publication of 755 cases of PORP, along with data from four publications from KPK province provide a significant insight on DDH demographics, prevalence, geographic distribution and cumulative outcome of treatment provided. The PORP report will provide pathway for establishment of MSK screening protocols and encourage for further investigation on a wide era of parameters of DDH to achieve best possible outcome with earliest treatment.
